# A new vision of photothermal therapy assisted with gold nanorods for the treatment of mammary cancers in adult female rats†

**DOI:** 10.1039/d3na00595j

**Published:** 2023-12-04

**Authors:** Hend Gamal, Walid Tawfik, Hassan IH El-Sayyad, Ahmed N. Emam, Heba Mohamed Fahmy, Heba A. El-Ghaweet

**Affiliations:** a Department of Zoology, Faculty of Science, Mansoura University Mansoura Egypt; b National Institute of Laser Enhanced Sciences (NILES), Cairo University Cairo Egypt; c Refractories, Ceramics and Building Materials Department, Advanced Materials Technology & Mineral Resources Research Institute, National Research Centre (NRC) El Bohouth St. Dokki Cairo Egypt; d Department of Biophysics, Faculty of Science Cairo University Cairo Egypt; e Nanomedicine & Tissue Engineering Research Lab, Medical Research Centre of Excellence, National Research Centre El Bohouth St., Dokki 12622 Cairo Egypt

## Abstract

Over the past decade, the therapeutic landscape has markedly changed for patients with breast cancers (BCs), yet few studies have evaluated the power of the photothermal therapy (PTT) technique. The present study aimed to assess the potency of 7,12-dimethylbenz[*a*]anthracene (DMBA)-induced mammary cancer treatment with this technique. In total, forty-two adult virgin female Wistar rats were categorized into seven groups, negative control, polyvinylpyrrolidone-capped gold nanorods (PVP-AuNRs) positive control (400 μL per rat ∼ 78 ppm), NIR laser irradiation 808 nm positive control with an intensity of (808 nm NIR CW diode laser, 200 mW cm^−2^ for 5 min), DMBA-treatment, DMBA-induced mammary cancer group treated with polyvinylpyrrolidone-capped gold nanorods, DMBA-induced mammary cancer group treated with NIR laser irradiation, and DMBA-induced mammary cancer group treated with polyvinylpyrrolidone-capped gold nanorods and NIR laser irradiation. Treatment with polyvinylpyrrolidone-capped gold nanorods and/or NIR laser irradiation was performed after three weeks of DMBA-induced mammary cancer. The mammary tumor lesions in the rat model induced with DMBA are highly invasive. Synthesis and characterization of gold nanorods (AuNRs) with an aspect ratio ranging from 2.8 to 3 were employed to validate the nanostructure and polyvinylpyrrolidone capping and their stability in absorbing near-infrared light. As a result, the therapy strategy, DMBA + PVP-AuNRs + NIR, effectively treated the tumor and halted its growth. The mammary glands were dissected and subjected to biochemical analysis for serum and tissue. Our treatment technique improved the histological aspects of mammary cancer in various forms of mammary cancer detected. Immuno-histochemical localization and TEM images supported these results reflecting the efficacy of this technique. Finally, our findings uncover for the first time the revolutionary effect of the PTT strategy using PVP-capped AuNRs in selectively destroying mammary cancer cells in rats.

## Abstract 2

Graphical abstract showing the whole photothermal process assisted with gold nanoparticles in female rats.

## Graphical abstract

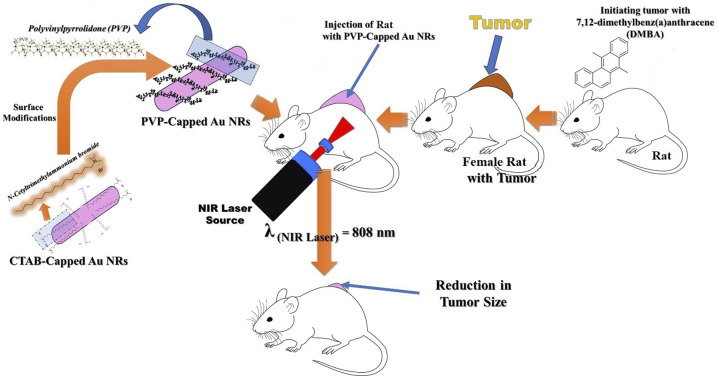

## 1. Introduction

Breast cancer (BC) is the most common cause of death in women. BC developed over several steps involving several cell types, and prevention is still a global challenge.^1^ Most women and men who develop BC are in their middle and later years. It is one of the worst diseases for women, which can spread to the body's key organs, including the lungs and bones. During their lifetime, one in eight women will develop BC.^2^ Recent big clinical trials for cancer, with some notable exceptions, have been unable to discover significant changes in treatment outcomes despite advances in basic research that have improved our understanding of tumor biology and helped to build new generations of targeted therapies.^3,4^ A polycyclic aromatic hydrocarbon (PAH) notorious for stressing out living things is 7,12-dimethylbenz[*a*]anthracene (DMBA). Petroleum and its derivatives and other organic pollutants, such as PAHs, are frequently released into the environment due to oil spills and incomplete combustion of fossil fuels.^5^ Rats exposed to DMBA develop mammary tumors, well-known after the mammary ‘glands’ metabolic activation. Ovariectomy reduces this model's susceptibility to DMBA, indicating that ovarian hormones are necessary for the carcinogen's inducible activity. The carcinogen's metabolites interact with the terminal end buds' rapidly proliferating cells, causing DNA adducts and mutations that contribute to the cells' development into cancerous cells.^6^ DMBA is a highly lipophilic molecule. Fatty tissue in the mammary glands allows DMBA to concentrate at epithelial contact before metabolic activation. This trait undoubtedly explains why DMBA is more active at the mammary level. In the phototherapy method known as photothermal therapy (PTT), laser irradiation can activate a substance called a photosensitizer (PS) and cause the creation of reactive oxygen species (ROS) molecules, which cause the malignant area to be destroyed.^7^ The discovery that PTT may trigger apoptosis raises the fascinating question of whether the intrinsic or extrinsic mitochondrial mechanism of apoptosis is the predominant pathway for cell death. Photosensitizers are commonly used in photodynamic treatment (PDT) because of their ability to create singlet oxygen by light absorption at the peak wavelength of PS.^8^ Today, the successful integration of photodynamic therapy with nanoparticles (NPs) has demonstrated significant advancements in the treatment process.^9^ Deep tissue penetration of NPs increases the improved permeability and retention (EPR) effect. When used in treatment, gold nanoparticles (AuNPs) are less likely to cause side effects than conventional medicines.^10^ Gold nanorods (Au NRs) can thus be broadly applied in biomedicine, particularly in cancer treatment. The surface functionalization of Au NRs significantly impacts reducing the toxicity of residual cetyltrimethylammonium bromide (CTAB).

The rationale for choosing gold nanoparticles (AuNPs) with different hetero-nanostructures gained great attention to be used in photothermal therapy due to their unusual optical-electron characteristics, which vary with their sizes and shapes. In this regard, surface plasmon resonance (SPR) occurs when free electrons resonate at the metal's surface at a specific frequency of incident light. The oscillation nonradiative decay, which converts light energy to heat, can thus cause greater light absorption and provide increased photothermal conversion efficiency.^11^ For example, Au NRs are anisotropic and tenable, which means their optical and chemical characteristics change depending on the orientation and synthesis factors. In addition, Au NRs are characterized by the feature of two SPR bands: one longitudinal that fluctuates with the aspect ratio and one transverse that is rather constant. Furthermore, Au NRs have strong visible-NIR light absorption, and the longitudinal peak redshifts as the aspect ratio increases.^11,12^ Incident light with NIR wavelengths is typically chosen because it penetrates deeply into the body and is rarely absorbed and scattered before reaching the Au NRs moderate temperature rise in the target region is required to selectively damage tumor tissues, which are more sensitive to hyperthermia than healthy tissues.^11–13^ Moreover, Au NPs showed lower toxicity in healthy cells than other metallic nanoparticles such as silver nanoparticles (Ag NPs) or nanoshells such as gold-coated silver nanostructures (Au–Ag NSs) due to its noble properties; in addition, the toxicological nature of Ag NPs may be attributed to the presence of Ag_2_O in both Ag NPs and Au–Ag NSs that release Ag^1+^ ions causing disruption of cells and generation of harmful ROS.^14,15^

Polyvinylpyrrolidone (PVP) is considered one the most important and common capping agents used in the preparation and controlling size and shape of nanomaterials; in addition, it overcomes problems associated with traditional nanoparticle manufacturing processes, such as toxicity and aggregation. As a result, PVP is used to create eco-friendly nano-formulations with greater application.^16^ However, little is known about how AuNP stabilizers affect the ability of endothelial cells (ECs) to survive and the performance of healthy blood vessels. Identifying gold nanoparticles, which can efficiently produce heat with low-energy light in response to wavelengths, may mark a significant turning point in developing photothermal therapy for cancer treatment. Near-infrared (NIR) rays can reach the mammary tissue about 10 cm deep.^17^ Water, blood, melanin, fat, and yellow pigments are vascularized tissue's main chromophores. They have a lower absorption coefficient in the NIR region, between 800 and 1200 nm. The “therapeutic window” is the name given to this band of wavelengths.^18^ NIR light-mediated photothermal therapy is a potential technique for treating surface and deep-seated cancers because of its high tissue permeability and low injury to healthy tissues. Furthermore, from a technological standpoint, therapy using the PTT approach results in effectiveness, convenience of handling, and cheap cost. It also has very minimal drug–drug interactions.^19^

Herein, polyvinylpyrrolidone-capped gold nanorods (PVP-capped AuNRs) have been prepared *via* a seed-mediated method for NIR light-mediated photothermal therapy of breast cancer. The effect of polyvinylpyrrolidone as capping on the particle size (*i.e.*, aspect ratio “R”), optical properties, and colloidal properties of gold nanorods (AuNRs) have been characterized either in the presence or absence of PVP—furthermore, the therapeutic effect of PVP-capped AuNRs against breast cancer either before or after exposure to NIR light. Moreover, the therapeutic assessment included the physiological parameters such as body weight, mammary tumor development, and morphometric changes; biochemical markers in serum, histopathological, and immunohistochemistry of mammary glands have been investigated.

## 2. Experimental

### 2.1 Materials

Hydrogen tetrachloroaurate(iii) trihydrate (HAuCl_4_·3H_2_O, LOBA chemicals, 99.999%). Polyvinylpyrrolidone (PVP, 30 K, 99%), 7,12-dimethylbenz[*a*]anthracene (DMBA, 99%), and dimethyl sulfoxide (DMSO, D2560, 99%) were purchased from Sigma-Aldrich. Sodium borohydride (NaBH_4_, sd-fine chemicals 98%), *N*-cetyl *N*,*N*,*N*,-trimethyl ammonium bromide (CTAB, Merck, 99%), silver nitrate (AgNO_3_, Sigma Aldrich, 99%), l-ascorbic acid (vitamin C, 99%), hydrogen peroxide (H_2_O_2_, 50%) and xylene were purchased from El-Nasr. phosphate buffer saline (PBS) was purchased from GeneTech, purified water with a resistivity of 18 MW from the Millipore MilliQ water purification system.

### 2.2 Methodology

#### 2.2.1 Preparation of PVP-capped gold nanorods (PVP-capped AuNRs)

The seed-mediated approach was used to create colloidal CTAB capped-AuNRs, as previously reported by Emam *et al.*^20^ and Nikoobakht and El-Sayed.^21^ Briefly, seed solution was prepared through a chemical reduction of a mixture from an aqueous solution containing 2.5 mL of 1 mM gold ions (*i.e.*, HAuCl_4_·3H_2_O, Au^3+^ ions) and 2.5 mL of 0.2 M of CTAB under vigorous stirring using 0.6 mL of ice-cold 0.01 M of NaBH_4_ as a mild reducing agent—a reddish-brown colloidal solution formed upon the dropwise addition of NaBH_4_, indicating the formation of gold nanoclusters. The seed solution was then aged for 8–12 minutes with vigorous stirring before being inserted into the growth solution to create AuNRs.

A 100 mL growth solution was generated by vigorously swirling 1 mL of an aqueous AgNO_3_ solution into 50 mL of 0.2 M CTAB. Then, 50 mL of 1 mM HAuCl_4_·3H_2_O (*i.e.*, Au^3+^) was added vigorously until a yellowish-brown solution was formed. Finally, a sufficient volume of 0.1 M l-ascorbic acid aqueous solution was added to the mixture with moderate shaking until a yellowish-brown hue faded, indicating the creation of an Au^1+^ solution. A sufficient volume of 1.2 mL of Au^0^ seed solution was promptly put into the growth solution to manufacture NIR-absorbed gold nanorods to form CTAB-capped AuNRs with the required aspect ratio. For around 60 minutes, the reaction medium was left alone. The solution's color changed with time, showing the development of CTAB-capped AuNRs. CTAB capped-AuNRs were cleaned from excess CTAB by centrifugation at 14 000 rpm for 10 minutes at 10 °C. The pellets were then re-dispersed into distilled water.

The surface-modified gold nanorods containing PVP were created using the approach described by Requejo *et al.*^22^ In this procedure, 5 g of PVP (30 K, 0.05 mg mL^−1^) was mixed with as-purified gold nanorods for 120 minutes before being left at room temperature overnight.

#### 2.2.2 Characterization

The photophysical properties include the UV-vis absorption spectra of the as-prepared CTAB capped- and PVP capped-AuNRs using TG-80 UV-vis double-beam spectrometer (PG instrument. Ltd., England). The absorption spectra were recorded at a range from 200 to 900 nm with an increment of 5 nm. In addition, the morphological properties, such as particle size and shape, have been investigated using a high-resolution transmission electron microscope (TEM) JEOL JEM-2100, Japan) operating at a voltage of 160 kV. The crystallographic structure of as-prepared gold nanorods (AuNRs) investigated *via* the XRD were recorded using Panalytical X'Pert Pro, Cu Kα radiation, (*λ* = 1.546 Å) over a 2*θ* range of 4–80°, with step 0.02°. Also, the colloidal stability properties, including the demonstration of hydrodynamic diameter (*H*_D_) and zeta-potential based on dynamic light scattering techniques, have been investigated using the Malvern zeta sizer Nano ZS Nano instrument with He/Ne laser (*i.e.*, *λ* = 633 nm) at 173° collecting backscatter optics. The colloidal properties for each as-prepared CTAB capped-AuNRs and PVP capped-AuNRs samples were examined by adding a small adequate of each sample and dispersed into dist. H_2_O for 10 minutes under ultrasonication before measurements.

#### 2.2.3 Animal experiments and ethical guidelines

According to the Faculty of Science Mansoura University ethics committee, the Experimental Animal Ethical Committee of Mansoura University in Egypt approved using laboratory animals in this study following the National Institutes of Health guidelines, with ethical approval number “Sci-z-ph-2021-41”. The experiment was carried out at Egypt's Helwan Breeding Farm with forty-two virgin female Wistar albino rats with an average weight of 100 ± 10 g. They just had two weeks to be ready for the experiment. They had full access to food and drink and were well-ventilated thanks to a 12 hour light/dark cycle.

#### 2.2.4 Induction of mammary carcinogenesis

The induction of mammary carcinogenesis was performed using 7,12-dimethylbenz[*a*]anthracene (DMBA). The furthermost broadly used active chemical inductors of mammary carcinogenesis. The dose was prepared using 250 mg of DMBA powder dissolved in 1 mL DMSO and 24 mL olive oil, as reported by Hou *et al.*^23^ The applied dose was 10 mg per rat, and three dosages were injected subcutaneously thrice each week, with follow-up.^24^

### 2.3 Experimental animals

A total of 42 adult virgin female Wistar albino rats (*Rattus norvegicus*) weighing 100 ± 10 g were divided into seven groups (*n* = 6), including a negative control, PVP-capped AuNRs positive control group, NIR laser irradiation positive control group, DMBA carcinogenesis-treatment, DMBA induced mammary cancer treated with PVP-capped AuNRs, DMBA induced mammary cancer treated with NIR laser three weeks after DMBA-induced breast carcinogenesis, PVP-capped AuNRs (400 μL per rat ∼78 ppm) were administered in a single dosage. After the PVP-capped AuNRs injection, the therapy was followed by a five-minute NIR laser treatment at 808 nm NIR CW diode laser, 200 mW cm^−2^ for five minutes. PVP-capped AuNRs were injected intraperitoneally.

The tumor volume was calculated using the formula *V* = π/6(*L* × *W* × *W*), where *L* stands for the tumor's length and *W* for its width. The tumor size was measured using a digital calliper with 0.01 mm resolution. The animals had an additional week of therapy before being slain with 300 mg kg^−1^ of chloral hydrate for sacrifice.

#### 2.3.1 Body weight change and absolute and relative liver, heart, and lung weight

The absolute body weight changes were determined before (three weeks from induction carcinogenesis) and after treatment (one week from treatment). At the same time, the absolute and relative liver, heart, and lung weights were also recorded after one week of treatment.

#### 2.3.2 Biochemical assessments of serum hormonal levels

Serum was extracted from the blood samples and kept at 20 °C until it was used to determine the levels of the standard markers carcinoembryonic antigen (CEA), estradiol (ER), and progesterone (PGR). The estradiol was assayed at 450 nm using an ELISA kit from CUSABIO (catalogue no. CSB-E06848r). Carcinoembryonic antigen was assessed using an ELISA kit from Fine Test Company, and progesterone was measured using an ELISA kit from instruction manual (catalogue no. ER0492).

#### 2.3.3 Biochemical assays

The subjects' mammary glands were centrifuged after homogenizing the mammary glands with phosphate buffer. The supernatants were separated and refrigerated. ER, MUC 1, MMP, HSP-90, and NF-kB were measured using an enzyme-linked immunosorbent assay (ELISA) kit from CUSABIO at 450 nm (catalogue no. CSB-E13148r). Using an ELISA kit from CUSABIO, the estradiol was measured at 450 nm (catalogue no. CSB-E06848r). The ELISA kit from instruction manual measures cell surface-associated (MUC 1) at 450 nm (catalogue no. MBS2024481). ELISA kit (competitive ELISA) measured matrix metallopeptidase 9 at 450 nm (catalogue no. MBS722532). At 450 nm, heat shock protein 90 was measured using an ELISA kit from the instruction manual (catalogue no. MBS764361).

#### 2.3.4 Histological analysis

The samples (mammary glands and liver) were fixed in phosphate-buffered formalin (pH = 7.4) for 10 minutes, then dehydrated in ethyl alcohol of increasing strength, washed in xylene, and mounted in melted paraplast at 58–62 C. Hematoxylin and eosin were used to stain 5 m slices of histology tissue, then examined using a bright field light microscope.

#### 2.3.5 Ultrastructural examination

Additional samples were post-fixed in 1% phosphate-buffered osmium tetroxide after being fixed in 2.5% phosphate-buffered glutaraldehyde (pH = 7.4). The samples were dehydrated in stronger ethyl alcohol, cleaned in propylene oxide, and set in epoxy resin. Using an LKB Ultra tome IV, extremely thin portions were cut and then put on grids. They were observed using a Joel 100CXl transmission electron microscope after being treated with uranyl acetate and lead citrate (Mansoura University, Egypt).

#### 2.3.6 Immunohistochemistry for Bcl-2, caspase 3, GATA-3, and COX-2

Five-micrometer formalin-fixed, paraffin-embedded mammary gland samples were placed on polylysine-coated glass slides. Tissue samples were then dewaxed, developed in xylene, and rehydrated in a series of alcohols. Slices of tissue were exposed to 3% H_2_O_2_ for 10 minutes to reduce endogenous peroxidase activity. The tissue sections were incubated in digested medium containing 0.05% trypsin (pH = 7.8) for 15 min at 37 °C with the primary monoclonal mouse antibody against the anti-apoptotic protein (Bcl-2), caspase-3 (DAKO, clone MIB5, mouse), and the primary antibody against (GATA-3) and (COX-2) antibodies at 1 : 50. After being cleaned, the slides were incubated with a secondary biotin-linked anti-mouse antibody for 50 minutes at room temperature, followed by another 50 minutes with the streptavidin-peroxidase complex. The sections were then counterstained with hematoxylin after being washed and developed using diaminobenzidine hydrogen peroxide (DAKO). Hematoxylin was used to counterstain the brown nuclear or cytoplasmic labelling to demonstrate the immune response. As negative controls, sections were treated with 1% nonimmune serum phosphate buffer solution (PBS) solution. The histological slices were examined using an Olympus bright field light microscope and a digital Canon camera. Additionally, slides for image analysis were taken using an Olympus® digital camera placed on an Olympus® microscope with a 1/2× photo adapter and a 40× objective. On a computer with an Intel® Core 5® processor, the acquired photographs were processed using the video test morphological software (Russia), and the percentage area was calculated and noted.

### 2.4 Statistical analysis

The relationship between different investigated groups was illustrated using one-way ANOVA chosen on the SPSS package (version 25 IBM for Windows). The probability levels were selected as follows: non-significant (*p* < 0.05), significant (*p* ≤ 0.05), highly significant (*p* ≤ 0.01), and very highly significant (*p* ≤ 0.001). In case of significant differences, the multiple range comparisons (least significant difference) were selected from the *post hoc* tests (Tukey HSD) on the same statistical package to detect the variances between diverse groups.

## 3. Results

### 3.1 Synthesis and characterization of bare and PVP-capped gold nanorods

**Fig. 1:**
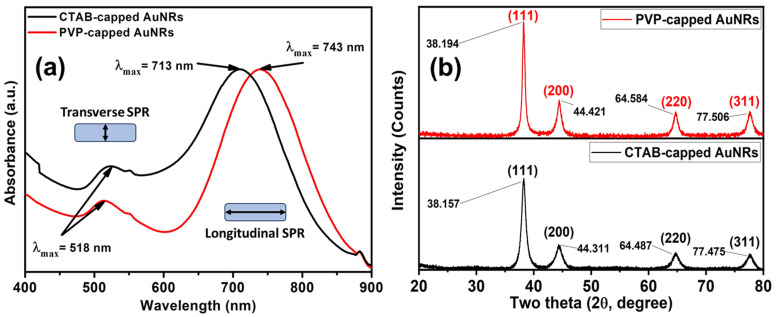
(a) UV-vis absorption spectra of CTAB (black-line) and PVP capped (red-line) Au-NRs (b) XRD patterns for both CTAB-capped and PVP-capped Au NRs.

CTAB capped-AuNRs were prepared through a three-step seed-mediated chemical recipe, as Emam *et al.*^20^ and Nikoobakht and El-Sayed^21^ reported. The first step is forming seed solutions from gold nanoclusters (Au^0^). A reddish-brown colour solution is formed *via* the chemical reduction of gold ions (Au^3+^) to tiny gold nanoclusters (Au^0^) in the existence of strong reducing agents such as BH_4_^−^ ions and CTAB as a surfactant. Whereas the second step was based on the formation growth solution; such solution is a colorless solution formed upon adding l-ascorbic acid (vitamin C) to the yellowish-brown solution of CTAB-Au^3+^. Where Au^3+^ ions are reduced to Au^1+^ due to the formation of [AuCl_2_]^−^ in the presence of silver ions (Ag^1+^), which have redox potential lower than Au^3+^ ions that facilitate the construction of gold nanorods *via* breaking the crystal symmetry in one direction than other directions/planes. Finally, the last step was adding an appreciated volume of seed solution to a growth solution to form Au-NRs (Fig. 1a) shows the optical absorption spectra of each CTAB capped- and PVP capped-AuNRs, respectively.

**Fig. 2:**
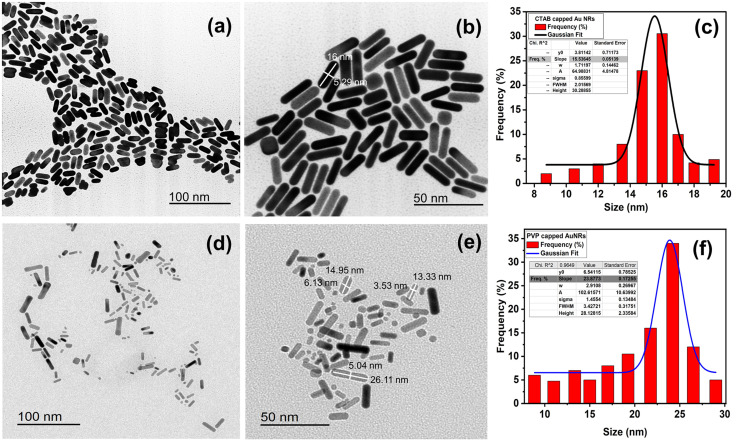
TEM images of (a and b) CTAB- and (d and e) PVP-capped Au-NRs. Frequency percentage % histogram of size distribution for CTAB capped and PVP capped AuNRs (c and f).

As illustrated in Fig. 1a, blackline, CTAB-capped AuNRs exhibited two distinct characteristics: the transverse surface plasmon resonance (T-SPR) at 518 nm and the longitudinal surface plasmon resonance (L-SPR) at 713 nm. While PVP-capped Au NRs exhibited a red shift in the L-SPR band in the absorption spectra compared to CTAB capped-AuNRs. The longitudinal surface plasmon band (L-SPR) in PVP capped-AuNRs was 745 nm, while CTAB capped-AuNRs had an L-SPR of 713 nm (see Fig. 1a, redline). Based on the UV-vis absorption spectra for both CTAB-capped Au NRs and PVP-capped Au NRs as shown in Fig. 1a. The red shift in the longitudinal surface plasmon band (L-SPR) is attributed to the change in the surrounding dielectric constant, which can affect on the aspect ratio of Au NRs (*i.e.*, length/width ratio) that aligned with the previous study.^25–28^ Furthermore, nanoparticle aggregation resulted in a considerable red-shifting of the SPR frequency, broadening of the surface plasmon band, and a change in solution colour from faint violet to dark violet colour due to interparticle plasmon interaction (*i.e.*, plasmon coupling).^28^ In such case, when the interparticle distance in the assembly falls below the average particle diameter, the electric dipole–dipole interaction and coupling between the plasmons of neighbouring particles in the assembly results in a bathochromic shift of the absorption band, which is known as the plasmon coupling band,^28,29^ which is confirmed by TEM images in Fig. 2e.

Also, the crystallographic structure of CTAB-capped and PVP-capped gold nanorods (AuNRs) showed is illustrated in Fig. 1b. As shown in Fig. 1b, the XRD pattern of CTAB-capped and PVP-capped Au NRs appeared to be identical. In CTAB-capped Au NRs, four distinct Bragg's reflections that characterized face-cantered cubic (FCC) crystal structure (111), (200), (220), and (311) were obtained at 38.157, 44.311, 64.487 and 77.475°, respectively. Upon the capping of Au NRs with PVP-30K same Bragg reflections are the same but with a slight shift to higher 2*θ* of 38.194, 44.421, 64.584, and 77.506° due to (111), (200), (220), and (311) reflections plans, respectively. In addition, a remarkable increase in the intensity of XRD patterns of PVP-capped Au NRs than CTAB-capped Au NRs due to increased stacking in the (200) and (220) planes as a result of the reorientation of the (111) planes in the dielectric polymeric medium of a relatively higher refractive index upon capping with PVP^30^ (See Fig. 1b). The calculated inter-planer distance was about 2.4, 2, 1.24, 1.4, and 1.2 Å, respectively.

Furthermore, the aspect ratio (*i.e.*, length-to-width “*R*”) of CTAB- and PVP-capped AuNRs was estimated using the Link *et al.* empirical formula.^25,26^ CTAB- and PVP-capped AuNRs have aspect ratios (*R*) of roughly 3.2 and 3.7, respectively. Furthermore, as shown in (Fig. 2a and b), the typical dimensions of CTAB capped-AuNRs are 16 ± 2.51 nm (length) and 5.33 ± 1.18 nm (width), with the associated aspect ratio ranging from 2.8 to 3. The typical dimensions of PVP capped-AuNRs were 19.25 ± 8.11 nm (length) and 4.71 ± 2.18 nm (width), with an aspect ratio ranging from 3 to 3.7, as shown in (Fig. 2d and e, respectively). Besides, the morphology of PVP-capped AuNRs changed so greatly compared to that of pure Au and this proven by statistical histogram about the percentage frequency % against the size of CTAB-capped and PVP-capped Au NRs (Fig. 2c and f, respectively).

**Fig. 3:**
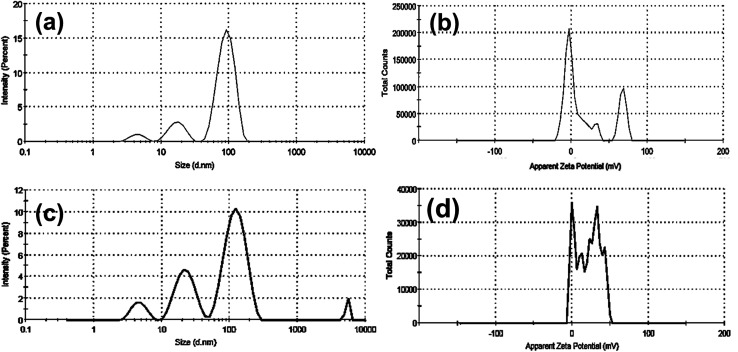
DLS and zeta potential data for both CTAB- (a and b), and PVP-capped (c and d) Au-NRs.

Furthermore, as shown in (Fig. 3a–d and Table 1), dynamic light scattering (DLS) and zeta-potential were utilized to investigate the colloidal stability of CTAB- and PVP capped-AuNRs. As demonstrated in (Fig. 3a and b, respectively), the CTAB capped-Au NRs had an average hydrodynamic diameter (*H*_D_) of about 93.12 nm, a polydispersity index (PDI) of 0.364, and a corresponding zeta potential (*η*) of around 23.3 mV. Upon the capping of Au NRs using polyvinylpyrrolidone (PVP), the average hydrodynamic diameter increased to 126.6 nm with a polydispersity index (PDI) ∼0.346, and the corresponding zeta potential (*η*) decreased to 18.3 mV, as shown in (Fig. 3c and d, respectively), which indicates that PVP-capped Au NRs have a low magnitude of zeta potential due to the steric hindrance created by the large PVP layer coating the surface of the CTAB-capped Au NRs. In addition, the cationic CTAB-capped AuNRs are electrostatically stabilized with the lone pair of electrons of oxygen in the carbonyl group and/or nitrogen in the pyrrolidone ring of the PVP molecule.

**Table 1:** Colloidal properties of both bare and PVP capped-Au NRs

Sample	DLS data			Zeta potential (mV)
	Hydrodynamic diameter (HD, nm)	The polydispersity index (PDI)	Z-average (nm)	
Bare Au NRs	93.12 ± 24.83	0.364	77.74	23.3
	17.45 ± 4.85			
PVP capped Au NRs	126.6 ± 42.01	0.346	74.86	18.3
	23.17 ± 7.56			

### 3.2. Body weight change and absolute and relative liver, heart, and lung weight

**Fig. 4:**
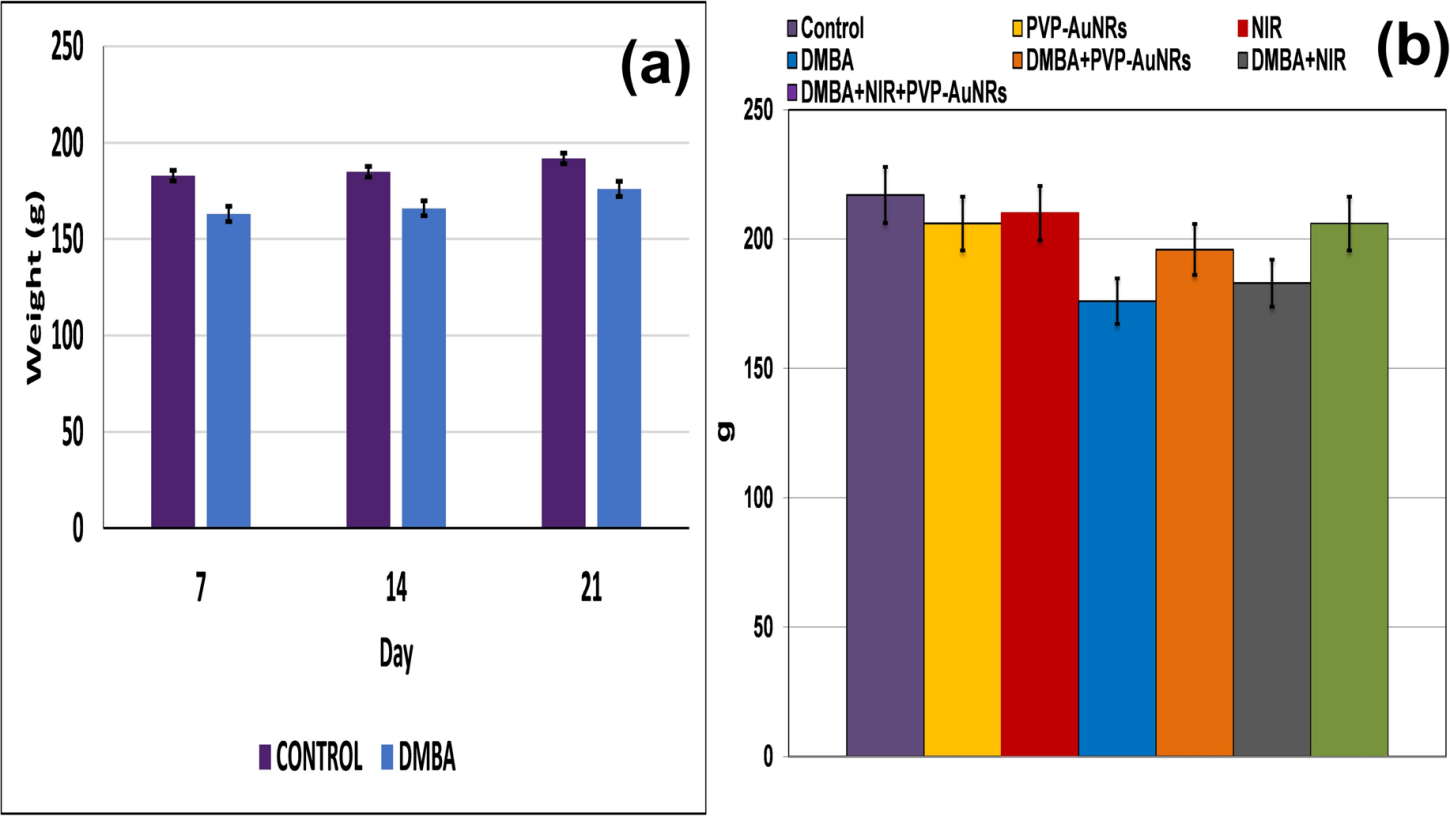
Change of body weight of female rats in control and DMBA carcinogenesis groups before treatment (a). Change of absolute body weight after 7 days from treatment (b).

**Fig. 5:**
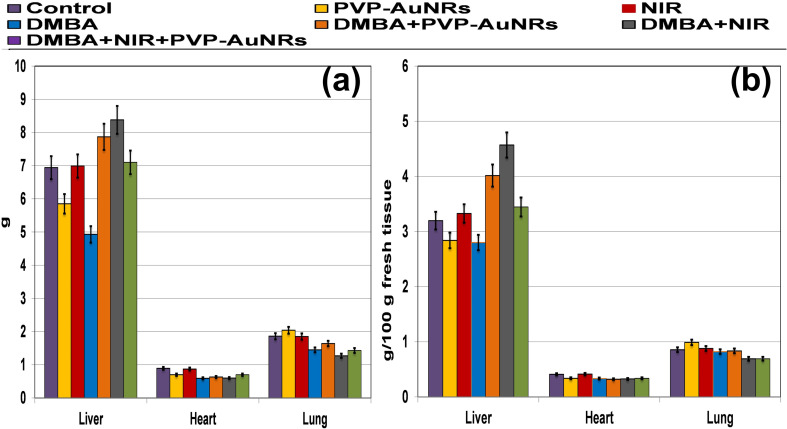
Both absolute (a) and relative (b) liver, heart, and lung weight of female rats subjected to DMBA carcinogenesis and irradiated with laser 808 nm intensity of 200 mV cm^−2^ for 5 min and/or PVP-capped AuNRs.

Compared to the control groups, the DMBA carcinogenesis group showed a depleted weight before treatment (Fig. 4a) (ESI: Table S1†). After treatment, the absolute body weight in the DMBA carcinogenesis group was markedly decreased compared to the laser-irradiated group. Laser irradiation treatment improved the DMBA group but was less amended than the PVP-capped AuNRs treated group. The weight increased significantly in the DMBA-induced mammary cancer group treated with PVP-capped AuNRs plus NIR laser irradiation compared to the control values (Fig. 4b). The DMBA-induced mammary cancer group treated with PVP-capped AuNRs plus NIR laser irradiation upregulated absolute and relative liver, heart, and lung weight, which varied markedly in other treated groups compared to the control values (Fig. 5a and b).

### 3.3 A schematic and actual demonstrations of mammary tumors development and morphometric changes in female rats subjected to DMBA carcinogenesis

**Fig. 6:**
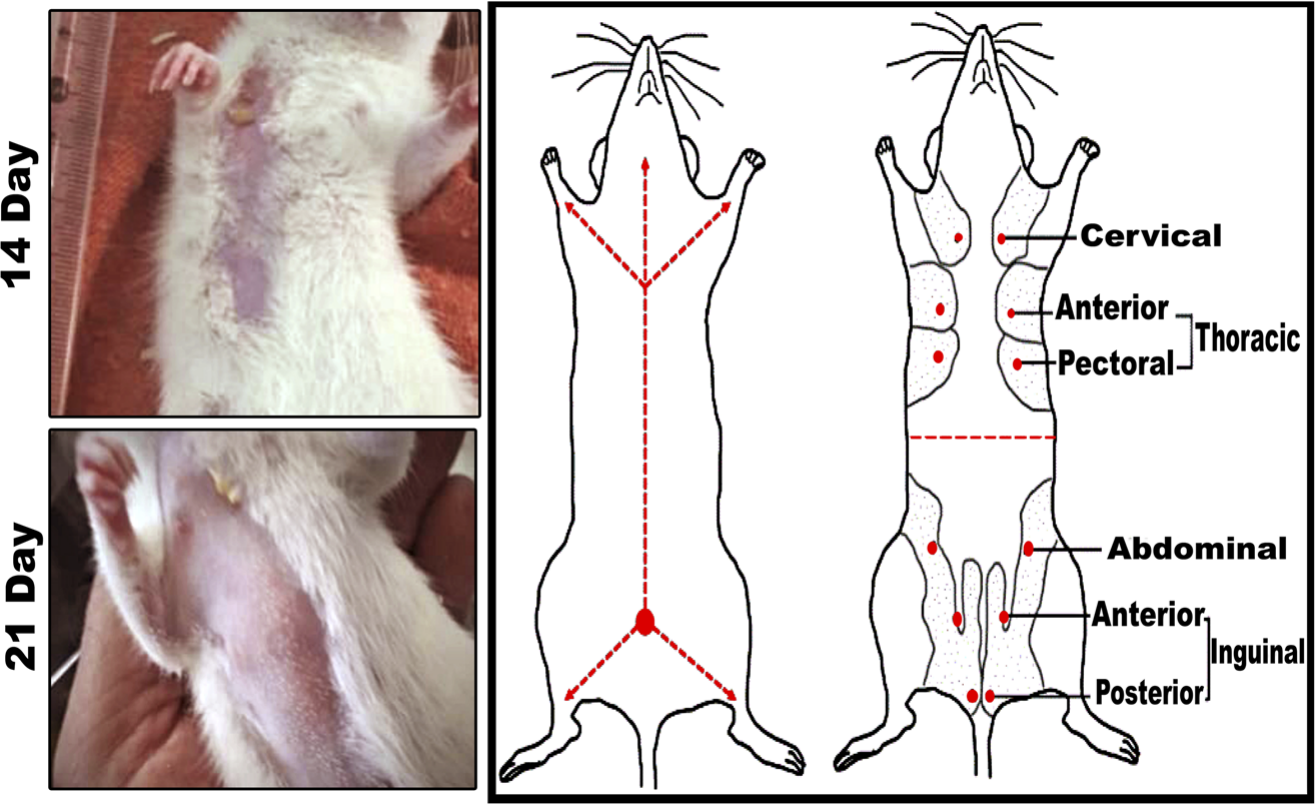
Mammary cancers induced by 7,12-dimethylbenz[*a*]anthracene (DMBA). (left panel) Tumor volume after 14- and 21-days of subjecting female rats to DMBA carcinogenesis. (right panel) Six ventral mammary gland pairs are present in the cervical, thoracic, abdominal, and inguinal areas of rats.

**Fig. 7:**
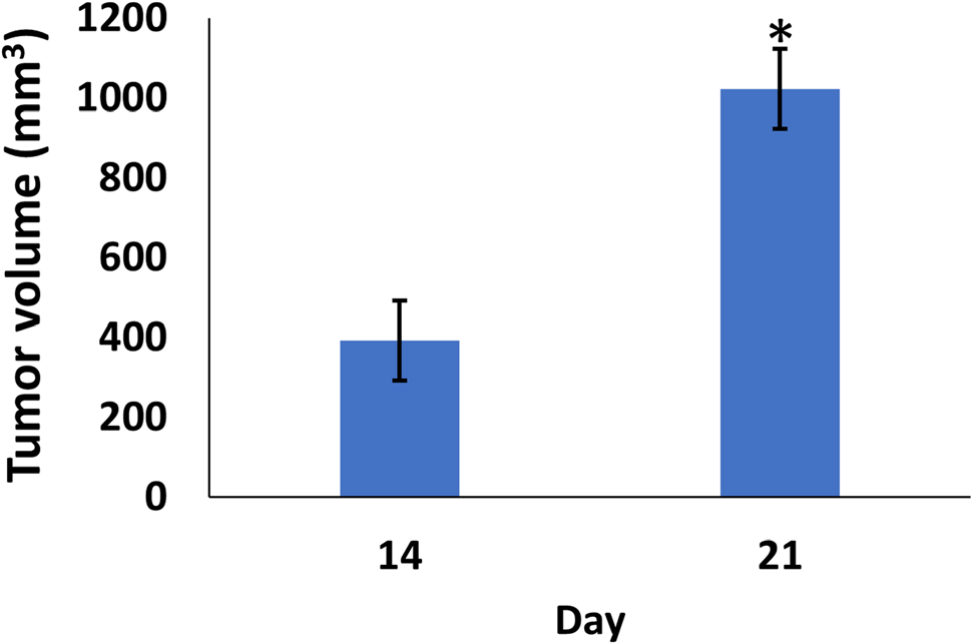
Change of tumor volume after 14- and 21-days of subjecting female rats to DMBA carcinogenesis. Each result represents the mean + S.E.M. (*n* = 6), * significant at *P* < 0.05.

In comparison to the control groups, the DMBA carcinogenesis group developed mammary cancers tumors 3 weeks after induction. On each side of the mammary glands, the tumor volume reached around 500 mm^3^ after the second week and more than 1000 mm^3^ during the third week (ESI: Table S2,†Fig. 6 and 7).

**Fig. 8:**
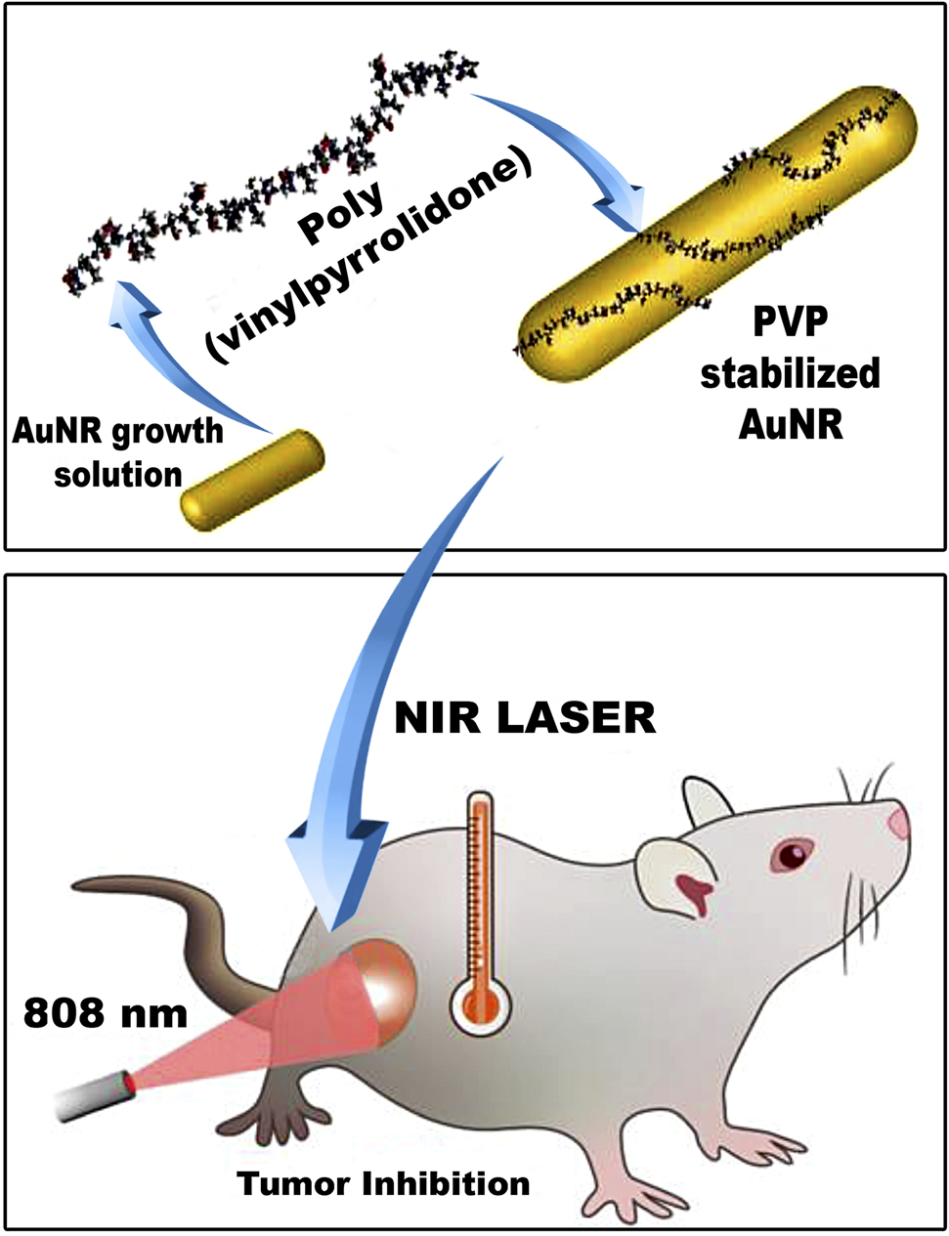
A schematic demonstration of treatment steps in the DMBA + PVP-AuNRs + NIR group, which is PVP-capped AuNRs injection (400 μL solution) intratumorally followed by NIR laser irradiation for 5 min.

**Fig. 9:**
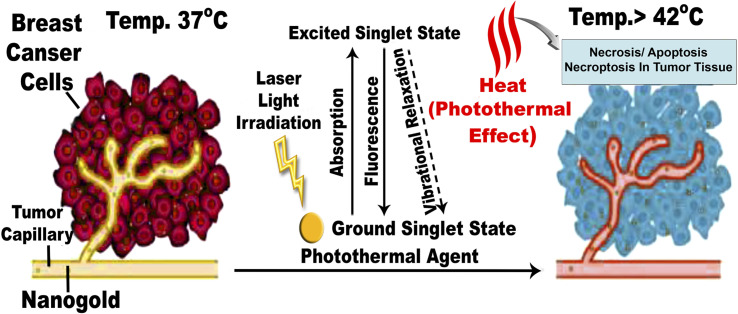
Mechanism of action for PTT effects in the breast tumor microenvironment mediated by nanogold. The EPR effect causes nanogold to accumulate inside solid tumors with leaky tumor vasculature. The nanogold has high absorption in the NIR window and is an effective heat transfer medium for laser energy. For tumor ablation, the photothermal effect is induced by the heat (>42 °C) produced during the vibrational relaxation of the excited PTT agents. This causes necrosis, apoptosis, and necroptosis of tumor tissue.^31^

**Fig. 10:**
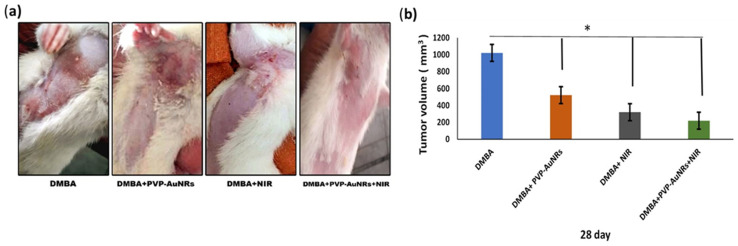
The real demonstration of cancer in cervical and thoracic mammary glands as an example and its size in four groups before and after treatment (a). Average tumor volume for different treatment groups over 1 week from treatment (b). Each result represents the mean + S.E.M. (*n* = 6), * significant at *P* < 0.05.

When DMBA carcinogenesis was treated with PVP-capped AuNRs + NIR laser irradiation, tumor volume was decreased by more than 75% relative to the untreated group (ESI: Table S3,†Fig. 8–10). In Fig. 9, we describe how nanogold mediates PTT activity in the mammary cancer microenvironment.

### 3.4 Biochemical markers in serum

The experimental DMBA carcinogenesis group demonstrated a considerable increase in CEA, estradiol, and PROG levels compared to the control group. On the other hand, CEA, estradiol, and PROG were reduced in the DMBA + PVP-AuNRs + NIR group after intra-tumor injection of PVP-capped AuNRs and 5 min of NIR laser irradiation. The degree of improvement, nonetheless, did not match that of the control (Table 2).

**Table 2:** Serum biochemical markers of breast tumors treated with PVP-capped AuNRs and/or laser irradiation^a^

Name of group	CEA (ng mL^−1^)	ER (pg mL^−1^)	PROG R (ng mL^−1^)
Control	0.77 + 0.03	96.25 + 0.3	4.12 + 0.3
PVP-AuNRs	0.71 + 0.02	93.52 + 0.5**	4.06 + 0.2
NIR	0.75 + 0.02	95.02 + 0.3	4.11 + 0.3
DMBA	4.85 + 0.3***	1002.67 + 0.7***	7.24 + 0.3***
DMBA + PVP-AuNRs	4.28 + 0.3***	862.41 + 0.5***	6.25 + 0.3***
DMBA + NIR	4.37 + 0.3***	855.92 + 0.6***	6.88 + 0.2***
DMBA + PVP-AuNRs + NIR	3.78 + 0.3***	689.94 + 0.5***	5.71 + 0.3**
*F*-Test	5.67	6.72	2.28
Sig	***	***	***

a Each result represents the mean ± SE (*n* = 6). * Means significant at *P* ≤ 0.05, ** means significant at *P* ≤ 0.01, *** means significant at *P* ≤ 0.001. CEA, carcinoembryonic antigen; ER, estrogen receptor; PGR, progesterone receptor; NIR, near-infrared; DMBA, 7,12-dimethylbenz[*a*]anthracene.

### 3.5 Biochemical markers in mammary glands tissues

Compared to the control groups, the experimental DMBA carcinogenesis group showed a significant increase in estradiol, MUC 1, MMP 9, HSP-90, and NF-kB contents. On the other hand, the DMBA + PVP-AuNRs + NIR group in which PVP-capped AuNRs were injected intra-tumor ally and followed by NIR laser irradiation for 5 min decreased estradiol, MUC 1, MMP 9, HSP-90 and NF-kB. However, the improvement degree did not match the control (Table 3).

**Table 3:** Biochemical markers of breast tumors treated with PVP-capped AuNRs and/or laser irradiation^a^

Name of group	ER (pg mg^−1^ protein)	MUC 1 (ng mg^−1^ protein)	MMP-9 9 (ng mg^−1^ protein)	HSP-90 (pg mg^−1^ protein)	NF-kB (pg mg^−1^ protein)
Control	170.58 + 0.5	0.628 + 0.03	1.52 + 0.2	217.25 + 0.3	4.86 + 0.3
PVP-AuNRs	171.05 + 0.3	0.617 + 0.03	1.48 + 0.1	214.21 + 0.3**	4.61 + 0.3
NIR	171.33 + 0.3	0.621 + 0.03	1.55 + 0.2	211.87 + 0.3***	4.37 + 0.2
DMBA	1050.92 + 0.6***	37.58 + 0.6***	10.89 + 0.4***	975.25 + 0.6***	8.94 + 0.4***
DMBA + PVP-AuNRs	877.52 + 0.6***	32.88 + 0.6***	8.15 + 0.3***	792.33 + 0.6***	7.39 + 0.3**
DMBA + NIR	892.41 + 0.6***	33.94 + 0.6***	9.02 + 0.3***	781.55 + 0.6***	7.21 + 0.3**
DMBA + PVP-AuNRs + NIR	705.44 + 0.6***	27.05 + 0.5***	7.82 + 0.3***	704.08 + 0.5***	6.97 + 0.5*
*F*-Test	6.09	1.59	2.06	4.95	2.21
Sig	***	***	***	***	***

a Each result represents the mean ± SE (*n* = 6). * Means significant at *P* ≤ 0.05, ** means significant at *P* ≤ 0.01, *** means significant at *P* ≤ 0.001. ER, estrogen R; L, laser; MUC 1, cell surface associated; G, gold; MMP 9, matrix metallopeptidase 9; HSP-90, heat shock protein 90; NFKB, nuclear factor kappa NIR, near-infrared; DMBA, 7,12-dimethylbenz[*a*]anthracene.

### 3.6 Histopathological observations of the female rat's mammary glands

**Fig. 11:**
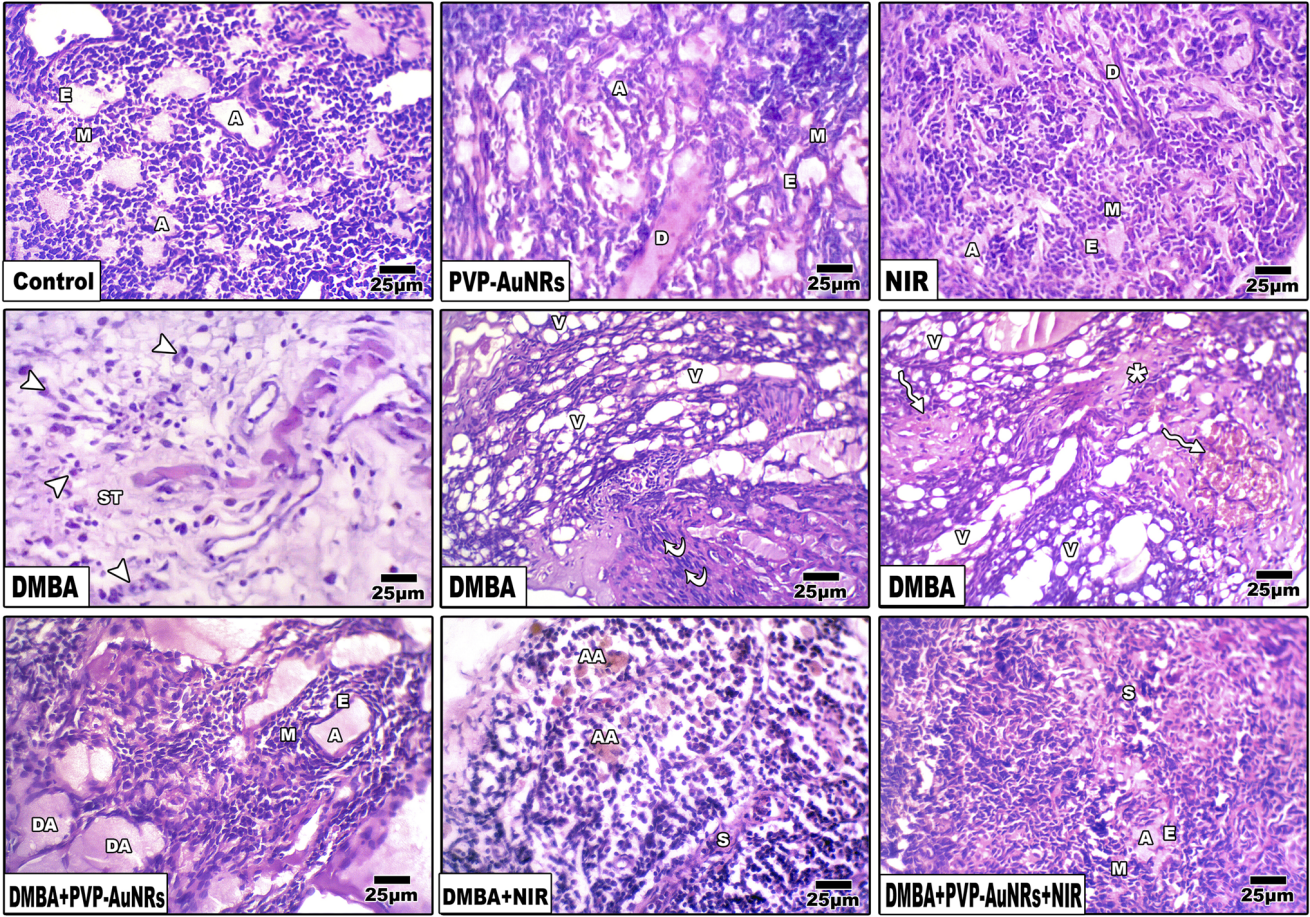
Photomicrographs of a histological cross-section of rat mammary glands respectively as follows. Negative control group. PVP-capped AuNRs positive control group. NIR laser irradiation positive control group. DMBA carcinogenesis showing, invasive ductal carcinoma (IDC), invasive lobular carcinoma (ILC), and lobule carcinoma *in situ* (LCIS) referred by arrowheads and invasive lobular carcinoma (ILC) referred by *. DMBA carcinogenesis treated with PVP-capped AuNRs. DMBA carcinogenesis treated with NIR laser irradiation. DMBA carcinogenesis treated with PVP-capped AuNRs and NIR laser irradiation. A, acinus; E, epithelial cell; M, myoepithelial cell; V, vacuole; S, septum. D, duct; ST, stroma; DA, dilated acini; AA, atrophied acini.

The epithelial bilayer was found to be made up of two different types of cells by the negative control group. The lactiferous ducts' inner section is made up of luminal cuboidal cells. Myoepithelial cells make up the bilayers' outside layer. These spindle-shaped cells help expel milk during mammary feeding. The mammary gland's epithelial components make up 10 to 15% of its total volume when combined (Fig. 11). Mammary glands of the PVP-capped AuNRs positive control group exhibited markedly dilated acini and pale stain in comparison to the control group (Fig. 11). The mammary glands of the NIR laser irradiation positive control group showed poorly differentiated acini with ducts in between (Fig. 11). Compared with the negative control, PVP-capped AuNRs and NIR laser irradiation positive control groups, the DMBA carcinogenesis group showed invasive ductal carcinoma (IDC) that takes place when altered milk duct lining cells infiltrate mammary tissue outside of the confines of the duct (Fig. 11), invasive lobular carcinoma (ILC) that starts in the mammary glands' lobules of milk-producing glands. Invasive cancer has spread outside the initial lobule it started in and can potentially spread to the lymph nodes and other body parts (Fig. 11). Lobule carcinoma *in situ* (LCIS), in which cells that resemble cancer cells develop in the lining of the mammary milk glands (lobules). However, they do not penetrate the lobules' wall, and invasive lobular carcinoma (ILC). In addition, vacuolation and supposed tumor cells were also detected among the intralobular supporting tissue (Fig. 11). DMBA carcinogenesis treated with PVP-capped AuNRs showed self-renewal and gland-reconstituting abilities. Numerous dilated acini were detected (Fig. 11). DMBA carcinogenesis treated with NIR laser irradiation showed a noticeable decline in ameliorative effect compared to the PVP-capped AuNRs treated group. A marked increase in atrophic acini was observed. In addition, the stromal element undergoes a myxoid degeneration, such as hyalinization of the intralobular tissue (Fig. 11). PVP-capped AuNRs and NIR laser irradiation treatment of the DMBA-carcinogenesis group showed regeneration of acini with intralobular septa. Signs of this improvement were the normal structure of acini and stroma-reconstituting abilities (Fig. 11). It is important to note that no metastases were found in the autopsy despite the tumor being malignant (see ESI†).

### 3.7 Histopathological observations of the liver

**Fig. 12:**
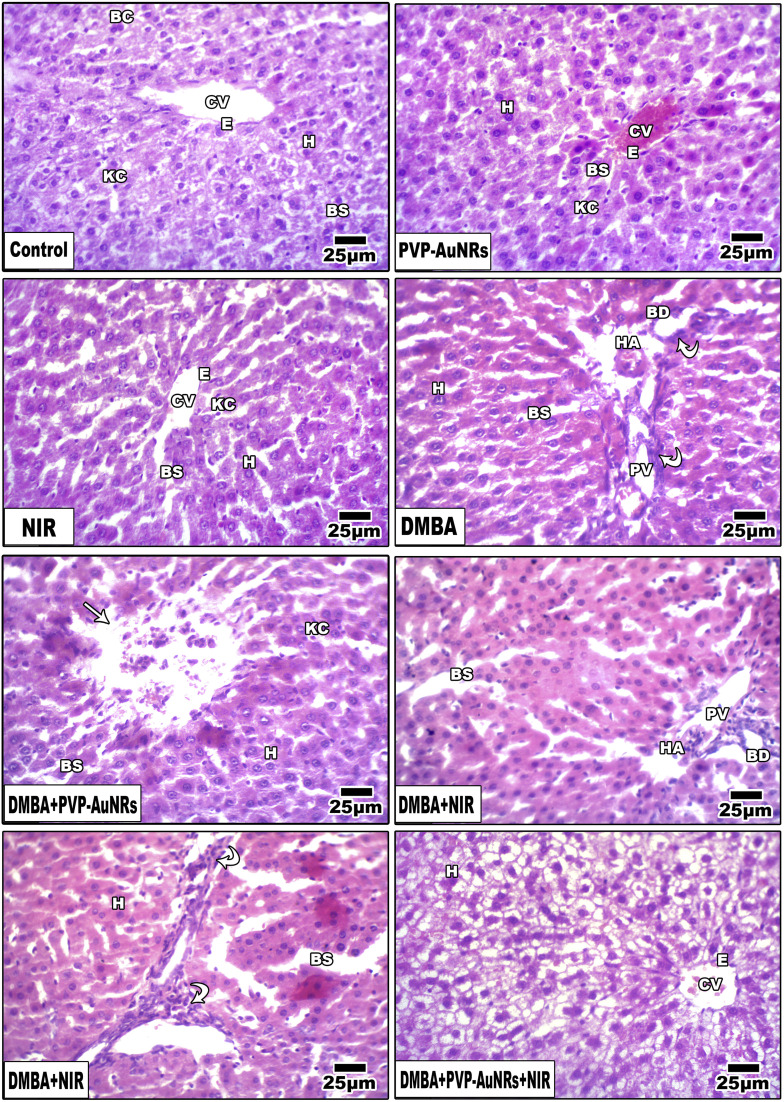
Photomicrographs of a histological cross-section of female rat liver. A, Negative control. PVP-capped AuNRs positive control group. NIR laser irradiation positive control group. DMBA carcinogenesis. DMBA carcinogenesis treated with PVP-capped AuNRs showing mononuclear cellular infiltration referred by arrowheads. DMBA carcinogenesis treated with NIR laser irradiation. DMBA carcinogenesis treated with PVP-capped AuNRs and NIR laser irradiation. PV, terminal portal venule; HA, hepatic artery; E, epithelial cell; KC, Kupffer cell; H, hepatocyte; BS, blood sinusoid; BD, bile ductule; CV, central vein. BC, binucleate cell.

Hepatic lobules made of cords of hepatocytes spreading out from the terminal hepatic venule were visible in histological sections of the liver parenchyma group of the negative control female rats. Narrow hepatic sinusoids coated with macrophages and specialized fenestrated endothelial cells separated these cell cords (Kupffer cells). A portal region of thin connective tissue, including branches of the hepatic portal vein, hepatic artery, and bile ducts, was seen around each lobule (Fig. 12). Liver of the PVP-capped AuNRs positive control group exhibited a maintained structure with a standard layout and pale stain. Most hepatocytes appeared normal, although congestion of hepatic sinusoids (Fig. 12). The liver of the positive control group irradiated with NIR laser irradiation positive control group showed dilation of blood sinusoids (Fig. 12). Compared with the negative control, PVP-capped AuNRs and NIR laser irradiation positive control groups, the DMBA carcinogenesis group showed an abnormal membrane of the portal vein, congested hepatic artery, degenerated bile duct, and dilated blood sinusoids. Bile duct hyperplasia and epithelium displacement were found (Fig. 12). DMBA carcinogenesis treated with PVP-capped AuNRs showed irregular architecture and lymphocyte cellular infiltration (Fig. 12). DMBA carcinogenesis treated with NIR laser irradiation showed disorganization of hepatic architecture and abnormal portal tract with congestion of portal vein and dilated blood sinusoids; scattered lobular inflammatory cells infiltrate (Fig. 12). PVP-capped AuNRs and NIR laser irradiation treatment of DMBA-carcinogenesis group showed an organization that maintains architecture, vacuolated hepatocytes with a regular central vein (Fig. 12) (see ESI†).

### 3.8 Immunohistochemistry of rat mammary gland

**Fig. 13:**
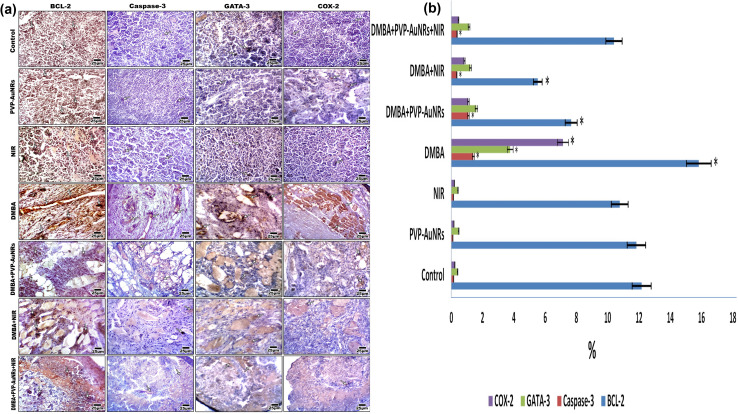
Photomicrographs of formalin-fixed mammary gland immunohistochemical stain with BCL2, caspase-3, GATA-3, and COX-2 vertical. The degree of immunoreactivity is shown by the arrowheads (a). Image analysis of female mammary glands subjected to DMBA carcinogenesis and treated with PVP-capped AuNRs and/or NIR laser irradiation. Images were taken under confocal microscopy at 400× magnification (b).

The DMBA carcinogenesis group displayed a much higher immunohistochemical response than the negative control group, PVP-capped AuNRs, and NIR laser irradiation positive control groups, which all had a moderate expression of BCL-2. However, the NIR laser irradiation group and the DMBA carcinogenesis treated with PVP-capped AuNRs both displayed a modest reduction in BCL-2 immunostaining (Fig. 13a). The proportion of immunostaining activity was significantly decreased in both the DMBA plus PVP-capped AuNRs-treated group and the DMBA plus NIR laser irradiation-treated group, according to image analysis of the BCL-2 (Fig. 13b) (see ESI†). The mammary gland cells that had undergone DMBA carcinogenesis and intoxication with DMBA + PVP-capped AuNRs showed upregulation of caspase-3, which indicates cell death. The immunostaining of caspase-3 was enhanced by co-administration of NIR laser irradiation to the DMBA plus PVP-capped AuNRs group. In addition, the DMBA carcinogenesis mammary gland cells subjected to NIR laser irradiation showed decreased caspase-3 staining (Fig. 13a). Compared to the other investigated groups, image analysis revealed a significantly higher level of caspase-3 in the DMBA + PVP-capped AuNRs and DMBA carcinogenesis groups (Fig. 13b). The DMBA carcinogenesis group showed enhanced immunostaining for GATA-3 immunohistochemistry, a sign of increased mammary cancer metastasis. However, the immunohistochemical response to GATA-3 was diminished when PVP-capped AuNRs were also administered. However, NIR laser irradiation treatment reduced the immunoreaction of GATA-3 in the DMBA carcinogenesis and/or PVP-capped AuNRs-therapy (Fig. 13a). After image analysis, GATA-3 demonstrated a much higher immune response in the DMBA carcinogenesis group compared to the other research groups (Fig. 13b). The mammary glands in the DMBA carcinogenesis group overexpressed COX-2 compared to the control group. However, when PVP-capped AuNRs were also given to the DMBA carcinogenesis group, COX-2 expression was reduced. Additionally, after receiving PVP-capped AuNRs therapy, immunostaining decreased after receiving DMBA carcinogenesis and/or NIR laser irradiation treatments (Fig. 13a). DMBA carcinogenesis increased significantly compared to the other study groups, according to image analysis of COX-2 (Fig. 13b) (see ESI†).

### 3.9 Electron microscopy

**Fig. 14:**
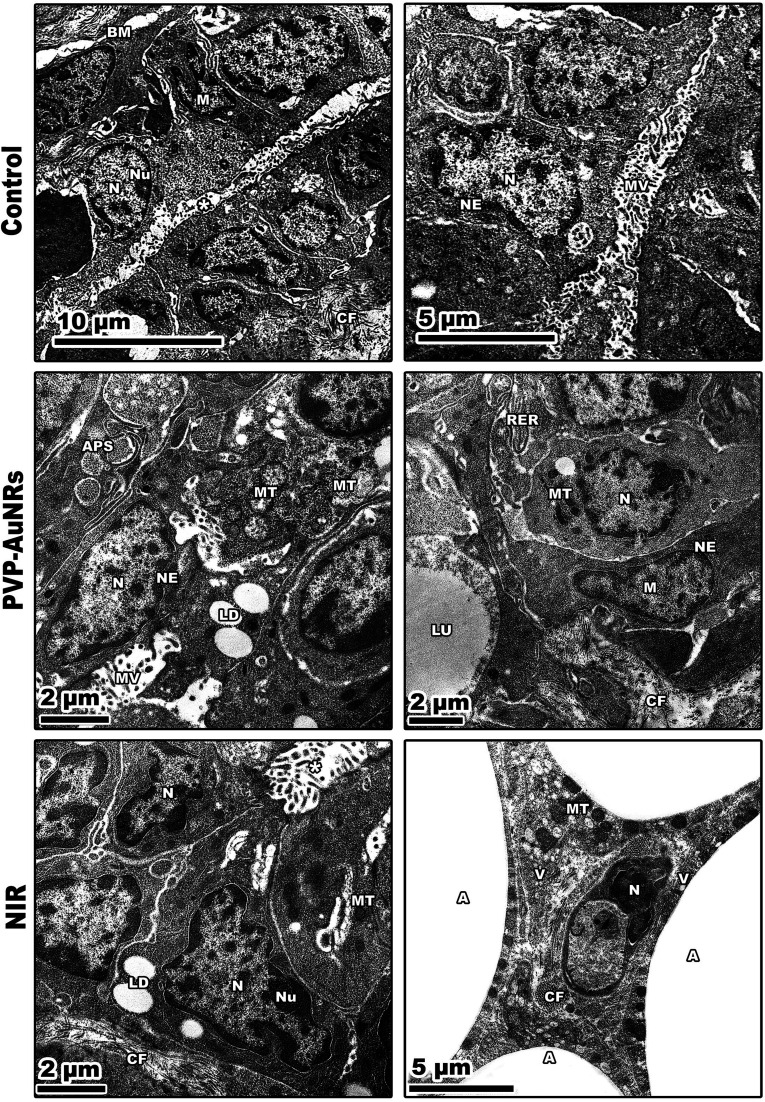
Negative control group showing two adjacent acini with their nuclei. PVP-capped AuNRs positive control group showing spindle shape myoepithelial cells with heterochromatic nuclei, and marked mitochondria associated-endoplasmic reticulum membranes (MAMs) with the formation of autophagosomes. NIR laser irradiation positive control group displaying a narrow, dense line along the nuclear envelope in the presence of nuclei with marginated heterochromatin. N, nuclei; M, myoepithelial cell; *, intracellular lumen. BM, basement membrane; NU, nucleoli; CF, collagen fibres; MV, microvilli; NE, nuclear envelope; LU, lumen; MT, mitochondria; LD, lipid droplets; APS, autophagosomes; RER, rough endoplasmic reticulum; A, adipose tissue.

Large, centred nuclei were a defining feature of many normal mammary gland cells. Acini are separated by luminal epithelial cells. The luminal face of the cells bore irregular short microvilli (Fig. 14). In the PVP-capped AuNRs positive control group, cell cytoplasm was filled with lipid droplets, and a few mitochondria interspersed in between the rER. In addition, marked mitochondria associated-endoplasmic reticulum membranes (MAMs) with the formation of autophagosomes were observed (Fig. 14). NIR laser irradiation positive control group showed a blood vessel with endothelial cell surrounded by a perivascular basement membrane between adipose tissue and numerous spherical mitochondria and vacuoles (Fig. 14) (see ESI†).

**Fig. 15:**
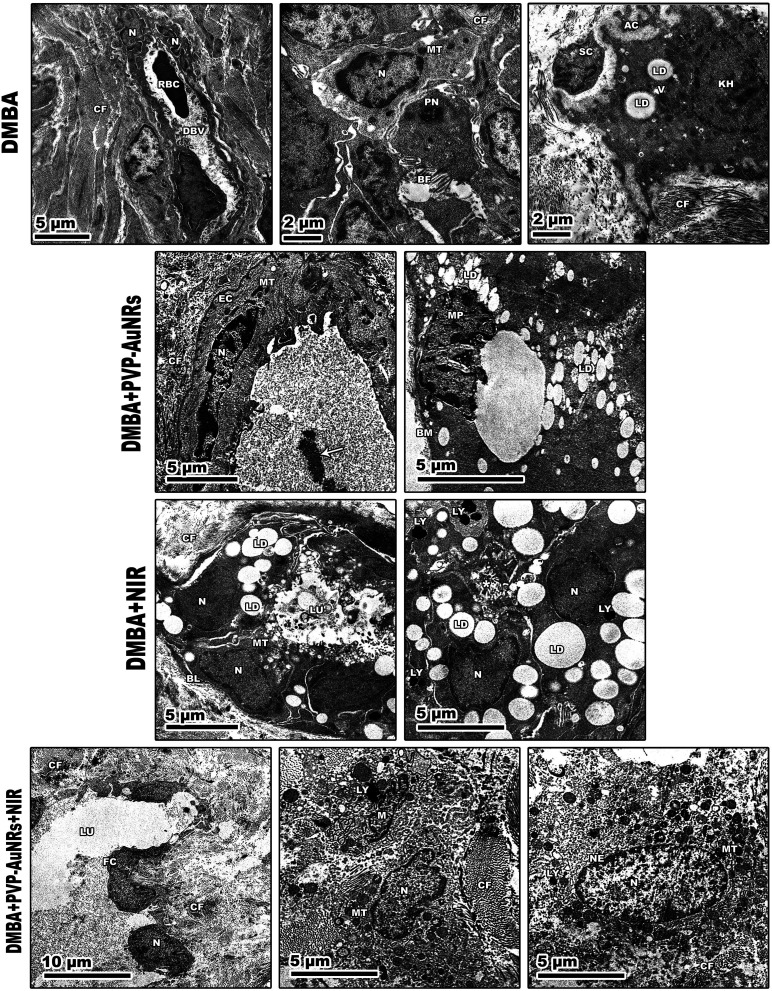
Transmission electron micrographs of rats' mammary tumors. DMBA carcinogenesis shows dilated blood vessels with nuclei of its endothelial cells and its perivascular basement membrane showing disorganization. Increased tumor collagen fibres formation. Pyknotic nucleus with chromatin condensation. Degenerated cancer cells showing: (1) cell shrinkage (2) apoptotic cell with cytoplasmic fragmentation (3) karyorrhexis nucleus. PVP-capped AuNRs treated group showing normal endothelial cell lines blood vessel with aggregation of PVP-capped AuNRs within the lumen (arrow). NIR laser irradiation-treated group showing breast lobule lining with epithelial and myoepithelial cells. DMBA carcinogenesis treated with PVP-capped AuNRs, and NIR laser irradiation group showing fibroblast cells with extensive cytoplasmic projections and collagen fibres deposition. Abbreviations: V, vacuoles; DBV, dilated blood vessel; RBC, red blood cell; BF, basal folding; PN, pyknotic nucleus; AC, apoptotic cell; KH, karyorrhexis nucleus; MP, macrophage cell; SC; shrinkage cell; FC, fibroblast cell; EC, endothelial cell; LY, lysosomes; BL, basal lamina.

The mammary tumors contained three distinct cell types: macrophages, cancer cells, and cancer cells that resembled mammary glands. While some mammary gland-like cancer cells were in various stages of degeneration, others were normal, as in the control samples. The DMBA carcinogenesis group showed dilated blood vessel and cell shrinkage and increased tumor collagen fibres formation compared to the control group. An advanced degree of cell degeneration was visible as an apoptotic cell with totally lysed organelles harbouring nuclear remains. Other cancer cells that had deteriorated showed karyorrhexis and pyknotic nuclei (Fig. 15). Regarding the post-treatment with PVP-capped AuNRs, the tumors-induced rats revealed immune cells with activity, including macrophages. Blood vessels with normal endothelial cells lining them and the appearance of gold particles inside them (Fig. 15). Mammary gland-like cancer cells and cancer cells that displayed degenerative alterations, such as prominent peripheral nuclear chromatin condensation, were present in the mammary tumors of rats exposed to NIR laser irradiation. A few scattered lysosomes and lipid droplets were detected (Fig. 15). However, DMBA carcinogenesis treated with PVP-capped AuNRs followed by NIR laser irradiation group showed fibroblast cells with extensive cytoplasmic projections and collagen fibres deposition. An unevenly shaped myoepithelial cell with prominent peripheral chromatin condensation, lysosomes, and lysed mitochondrial cristae (Fig. 15). The mammary gland cells have big, central nuclei, and their cytoplasm is pale because it lacks most of the usual cytoplasmic organelles, except for a few mitochondria and ribosomes. Compared to the control groups, advanced cancer cells have distinct peripheral nuclear condensation, vesicular-swollen mitochondria, and lysosomes (Fig. 15) (see ESI†).

## 4. Discussion

A clinically licensed, minimally invasive therapeutic method called photothermal therapy (PTT) can potentially kill cancer cells selectively. Photosensitizer, light, and oxygen are the three primary components of PTT.^32^ By directing the medication payload to the illness site and minimizing off-target action, nanoparticle-based drug delivery has made it possible to overcome limitations with the present therapeutic techniques.^33^ Gold nanoparticles are biologically inert substances with great plasticity, which are excellent for medical applications.^34^ Gold nanoparticles with rod-like shape that capped with PVP and have an aspect ratio (length: width) 3 : 1 can absorb near-infrared (NIR) (to which our body is transparent) and convert it into heat. The tumor microenvironment (TME) is influenced by tumor growth and possesses top physiological traits such as hypoxia, mild acidity, and vascular irregularity. Colloidal gold has been demonstrated to have localized plasmon surface resonance (LPSR), which means that gold nanoparticles can absorb light at wavelengths, producing photoacoustic and photothermal properties. These properties make colloidal gold useful for hyperthermic cancer treatments.^12^ Nevertheless, since there is too much cationic surfactant CTAB, the naked gold nanorods have a hazardous effect. PVP has thus been utilized as a biocompatible capping agent to replace CTAB through ligand exchange.^35,36^

All estimated photophysical and morphometric properties of PVP-capped AuNRs, like UV visible spectroscopy, TEM, DLS, and *η*, proved their validity and stability when entering the living cells in rats. PVP-capped AuNRs served as the PS in our primary treatment group (DMBA + PVP-capped AuNRs + NIR laser irradiation), which could be activated by NIR irradiation and generated singlet oxygen, which influenced PVP-capped AuNRs to cause hyperthermia. Injecting NPs into the mammary cancer rat model was followed by the NIR laser treatment was our strategy. In the current study, DMBA carcinogenesis considerably reduced overall weight gain and the weights of the liver and heart in absolute and relative terms. The *in vivo* findings showed that the induced tumors to more than 75% in the DMBA group underwent progressive growth during the whole treatment period. An average tumor volume reached more than 1000 mm^3^ before therapy. It continued to increase throughout the treatment, which suggests that if no treatment was used, the mammary tumors could grow significantly larger than their original size.^37^

Even though many studies have been conducted worldwide, only a few single biomarkers are useful in managing mammary cancer over time.^38^ Serum tumor markers for estradiol (ER), progesterone (PR), and carcinoembryonic antigen (CEA) were found to be higher in the DMBA group.^39^ The DMBA group showed a substantial increase in the selected mammary cancer indicators, indicating detrimental changes in mammary gland function. As a measure of sensitivity to endocrine therapy, ER (a) expression is, without a doubt, the most significant biomarker in breast cancer. Estradiol, a steroid hormone, is the primary growth factor for ER-positive tumors, accounting for 80% of breast cancers.^40^ MUC 1 and MMP 9, which served as predictive biomarkers for breast cancer patients, increased less as metastasis and invasion increased in breast cancer.^41–43^ Mammary ductal carcinomas have been found to have increased Hsp-90 expression, which promotes the proliferation of mammary cancer cells. Overexpression of Hsp-90 has been suggested as a part of the mechanism by which mammary cancer cells develop resistance to different stress stimuli.^44^ NF-κβ has a crucial role in controlling the proliferation and branching of mammary glands,^45^ and it also safeguards the epithelium during apoptotic alveolar involution. We and others have shown that NF-κβ factors are activated in mammary cancer: most main human breast and DMBA-induced rat mammary tumor tissues, breast cancer cell lines, and carcinogen-transformed human mammary epithelial cells contained significant levels of nuclear NF-κβ.^46^ Before the development of carcinogen-induced rat mammary tumors, NF-κβ up-regulation can occur.^47^

Our data supported the decrease and perceptible improvement in the DMBA plus PVP-capped AuNRs and NIR laser irradiation-treatment group for both tissue and serum tests. The chemical DMBA is extremely lipophilic. Adipose tissue in the mammary glands allows DMBA to concentrate at epithelial contact before metabolic activation. In our work, the mammary cancers induced by DMBA showed invasive ductal carcinoma (IDC), invasive lobular carcinoma (ILC), and lobule carcinoma *in situ* (LCIS). In addition, vacuolation and supposed tumor cells were detected among the intralobular supporting tissue. The results support that DMBA has a potent carcinogenic impact, particularly in SD rats.^48^ The mammary tissues were highly susceptible to PVP-capped AuNRs and NIR laser irradiation treatment, resulting in acini regeneration with intralobular septa. There was a normal structure of acini and stroma-reconstituting abilities. Also, we observed that no metastases were found in the autopsy despite the malignant tumor. In this regard, it is important to note that the liver, the organ where nanomaterials concentrate most, plays a crucial role in assessments of nanotoxicity.^49^

According to our results, the structural alterations in the hepatocytes (HC) of the DMBA-treated group indicated a detectable necro inflammatory. Unusual trabecular architecture and mononuclear cellular infiltration with pale stain were revealed by PVP-capped AuNRs treatment. These hepatocytes significantly improved in the PVP-capped AuNRs and NIR laser irradiation-treatment group. They showed a normal core vein with normal epithelial cells, surrounded by unremarkable-appearing hepatocytes that maintained their regular trabecular architecture and orientation. These findings corroborate those obtained by Gupta *et al.*, who reported that intra-tumor administered AuNRs, in conjunction with laser irradiation, effectively treated hepatocytes in animal models.^50^

We report that the combinatorial therapy strategy suggested here moderated the immunohistochemical reaction of caspase-3 and cyclooxygenase-2 (COX-2) compared to a significant increase in the DMBA carcinogenesis group, decreased the expression of the anti-apoptotic protein (BCL-2), and increased the immunohistochemistry of GATA-3 as a diagnostic marker for mammary cancer metastases.^51–53^ The TEM demonstrated the existence of tissue macrophages and lymphocytes linked to local dendritic cell (DC) activation and promoted DC migration to the lymphoid tissue containing their tumor antigens. The primary immunological response following treatment with PVP-capped AuNRs and exposure to NIR laser irradiation is observable from the TEM as macrophage phagocytoses. According to our research, mammary carcinomas' development is characterized by collagen fibres thickening, straightening, and aligning perpendicular to the tumor boundary in tandem with tumor invasion. When mammary tumors develop in mice, these modifications, known as “tumor-associated collagen signatures” (TACS), appear in certain ways.^54^ Increasing intermolecular crosslinks between collagen fibres may contribute to collagen fibre organization changes. During the development of tumors, lysyl oxidase, which catalysis collagen crosslinks, is enhanced.^55^ The extracellular matrix (ECM) is predicted to become stiffer because of crosslinking, which would impact mechano-signalling. Additionally, TEM micrographs showed that DMBA carcinogenesis led to the development of an aberrant basal folding project into luminal epithelial cells. The findings align with those obtained by Hinck and I. Näthke,^56^ who claimed that the creation of unpolarized cells in the mammary epithelium is no longer constrained by their intercellular contacts. These cells can migrate through the connective tissue and basement membrane, eventually breaching blood and lymphatic arteries to reach secondary sites. They can also remain bound in tiny clusters, undergo an epithelial-to-mesenchymal transition (EMT), and migrate singly. To help control the malignancies, the PVP-capped AuNRs restored cellular homeostasis and apoptotic responses in the cells. PVP-capped AuNRs activated the macrophage and autophagy processes, destroying permanent cancer cells.^57^

Additionally, low-level NIR laser irradiation therapy restored apoptotic reactions in cells by taking advantage of mitochondrial instability and generating ROS “bursts” that trigger apoptotic cascades in tumors with minimal harm to surrounding tissue.^57^ There were lots of cancer cells that looked like mammary glands. It was possible to trace the progression of degenerative alterations, starting with the rER swelling and progressing to nuclear shrinkage and mitochondrial cristae lysis. The TEM demonstrated how the typical cancer cells are affected by PVP-capped AuNRs and exposure to NIR laser irradiation, even if the mitochondria were initially unaffected. These enable the mammary gland-like cancer cells to self-renew and recreate glands. We demonstrated that AuNRs bioconjugate does not have harmful effects even at high concentrations, consistent with other research publications. Nevertheless, the delivery of decreased nanoconjugates or the bio-functionalization of AuNRs with biologic moieties may reduce toxicity, a significant barrier to the successful use of AuNRs in clinical applications.^58^

## Conclusions

In conclusion, it is generally known that the mammary tumors we employed as a model are overly aggressive because they have few available therapeutic choices. Considering this, our observations suggested that PVP-capped AuNRs and NIR laser irradiation therapy would be a promising alternative to chemotherapy for BCs and could enhance the therapeutic response in recurrent and early-stage disease variants. PVP-capped AuNRs and NIR laser irradiation treatment were able to cause mammary cancer degeneration and modify the immune responses against the highly invasive rat mammary carcinoma *in vivo*, according to research on the ultrastructure of rat mammary tumors. These findings let us understand that there is a good chance that this approach will replace the traditional and highly risky treatment modalities, which calls for additional supporting research.

## Ethics approval/consent to participate

All animal experiments in this study were approved by the Ethics Committee approval number “Sci-z-ph-2021-'41” according to the ethics committee at the Faculty of Science Mansoura University and were performed in accordance with the relevant guidelines and regulations.

## Data availability

All data generated or analyzed during this study are included in this manuscript.

## Author contributions

Hend Gamal (corresponding author); did the experimental cancer biology and animal studies practical work with the aid of Hassan IH El-Sayyad, and contributed to the formulation of the manuscript. Walid Tawfik provided chemicals used in this research and constructed the manuscript in its final form. Ahmed Nabile Emam; preparation and performing characterization investigation experiments for bare AuNRs and PVP capped AuNRs, including UV-Vis optical absorption spectroscopy, TEM, XRD, and dynamic light scattering (DLS) & zeta potential, data analysis, and contribution in the formulating and revision of the manuscript regarding the nanomaterials preparation and characterization in the final form before the submission. Heba Mohamed Fahmy; contributed to the conceptualization and the data analysis regarding the biological studies and contribution in constructing the manuscript. Heba Atef El-Ghaweet; categorized the data, investigating histopathology, ultrastructural, and immunohistochemistry, and revised the data.

## Conflicts of interest

There are no conflicts to declare.

## Supplementary Material

NA-006-D3NA00595J-s001
