# Supplementary material for: A new vision of photothermal therapy assisted with gold nanorods for the treatment of mammary cancers in adult female rats
Source: Nanoscale Adv. 2023 Dec 4;6(1):170–87. doi: 10.1039/d3na00595j (PMC10729923; doi:10.1039/d3na00595j)
Supplement: NA-006-D3NA00595J-s001 [file NA-006-D3NA00595J-s001.pdf]

Electronic Supplementary Material (ESI) for Nanoscale Advances.
This journal is © The Royal Society of Chemistry 2023

Title of data: datasets (Tables)

Additional information for Figures 4a, 5b, and 7b.

Description of data

Fig. 4a

Table S1: Change of body weight of female rats in control and DMBA carcinogenesis groups before treatment.

Days	Control	DMBA
**7**	183 + 3.4	163 + 3
**14**	185 + 5.9	166 + 4.6
**21**	192 + 4	176 + 3.9

Fig. 4b

Table S2. Change of tumor volume after 14- and 21- Days of subjecting female rats to DMBA carcinogenesis, each group composed of 6 rats (N=6).

			DMBA
**14 Day**	**Tumor Volume (mm^3)**	**Cervical & Thoracic glands**	392.5 + 2.93
**21 Day**			1022.65 + 1.33

Fig. 7b

Table S3. Change of tumor volume after 28- Days of subjecting female rats to DMBA carcinogenesis, each group composed of 6 rats (N=6).

			DMBA	DMBA+ PVP-AuNRs	DMBA+ NIR	DMBA+ PVP-AuNRs+ NIR
**28 Day**	**Tumor Volume (mm^3)**	**Cervical & Thoracic glands**	1022.65 + 2.06	523.6 + 1.05	321.56 + 1.55	220.89 + 2.05

Title of data: datasets (Text)

Additional information for a legend.

Description of data

Fig. 8

Photomicrographs of a histological cross-section of rat mammary glands. Negative control showing active acini with the lumen and basement membrane with myoepithelial cells. The lining epithelial cells vary from cuboidal to low columnar and contain cytoplasm, although septa of intralobular tissue remain. PVP-capped AuNRs positive control group showing active acini with pale staining due to PVP-capped AuNRs. NIR laser irradiation positive control group showing active acini with a duct. DMBA carcinogenesis showing invasive ductal carcinoma (IDC), invasive lobular carcinoma (ILC) showing vacuolated lobule, head arrows referring to invasive tumor cells, and lobule carcinoma in situ (LCIS) referred by arrowheads and invasive lobular carcinoma (ILC) referred by * exhibiting acini with vacuolation. DMBA carcinogenesis treated with PVP-capped AuNRs showing active dilated acini. DMBA carcinogenesis treated with NIR laser irradiation showing atrophied acini. DMBA carcinogenesis treated with PVP- capped Au NRs and NIR laser irradiation showing regeneration of acini with intralobular septa.

Fig. 9

Photomicrographs of a histological cross-section of female rat liver. Negative control showing the classical hepatic architecture. Blood sinusoids separate the hepatocytes, which are organized in cords emanating from the central vein which is lined with epithelial cells. PVP- capped AuNRs positive control group showing central vein, blood sinusoid, and Kupffer cell. NIR laser irradiation positive control group showing dilation of blood sinusoids. DMBA carcinogenesis shows an abnormal membrane of the portal vein, congested hepatic artery, degenerated bile duct, and dilated blood sinusoids. Note that the head of the arrows refers to inflammatory cells. DMBA carcinogenesis treated with PVP-capped AuNRs showing mononuclear cellular infiltration referred by arrowheads. DMBA carcinogenesis treated with NIR laser irradiation shows disorganization of hepatic architecture and abnormal portal tract with congestion of portal vein and dilated blood sinusoids. DMBA carcinogenesis treated with PVP-capped AuNRs and NIR laser irradiation showed vacuolated hepatocytes with a normal central vein.

Fig. 10 a

Observe the elevated immunostaining of BCL-2 in DMBA carcinogenesis and upgrading post-NIR laser irradiation of PVP-capped AuNRs and DMBA carcinogenesis. Caspase-3 intoxication showed an amplified immune reaction in PVP-capped AuNRs and DMBA carcinogenesis and enhanced in the NIR laser irradiation-treated studied group. GATA-3 showed overexpression in PVP-capped AuNRs and DMBA carcinogenesis and improved the NIR laser irradiation-treated studied group. COX-2 showed increased immunostaining in PVP-capped AuNRs and DMBA carcinogenesis and improved NIR laser irradiation-treated studied group.

Fig. 10 b

BCL-2 shows moderate image reaction in DMBA carcinogenesis, NIR laser irradiation, and/or PVP-capped AuNRs. Caspase-3 expressing apoptosis showed a notable increase in image analysis in DMBA carcinogenesis, NIR laser irradiation, and/or PVP-capped AuNRs. GATA-3 reflecting metastases showing a significant increase of image analysis in DMBA carcinogenesis and NIR laser irradiation and/or PVP-capped AuNRs. Image analysis in DMBA carcinogenesis, NIR laser irradiation, and/or PVP-capped AuNRs reveals an increase in COX-2 expressing inflammation.

Title of data: datasets (Text)

Additional information for a legend for Figure 5

Description of data

Fig. 11

Transmission electron micrographs of negative control, PVP-capped AuNRs, and NIR laser irradiation positive control groups of rats' mammary glands show Negative control showing two adjacent acini with their nuclei. Between each basement membrane and the secretory cells are the cytoplasmic processes of myoepithelial cells. Both are separated by luminal epithelial cells. The luminal face of the cells bore sporadic short microvilli. Nucleoli are obvious in some nuclei. The nuclear envelope of a nucleus with marginated heterochromatin shows up as a thin, dense line. PVP-capped AuNRs positive control group showing spindle shape myoepithelial cells with heterochromatic nuclei, a few spherical mitochondria with the rough endoplasmic reticulum, and marked mitochondria associated-endoplasmic reticulum membranes (MAMs) with the formation of autophagosomes. Notice the cytoplasm of the secretory cells contains lipid droplets and the presence of collagen fibers. NIR laser irradiation positive control group displaying a narrow, dense line along the nuclear envelope in the presence of nuclei with marginated heterochromatin. Nucleoli are obvious in some nuclei. Note also the presence of lipid droplets and luminal epithelial cells. A blood vessel with its endothelial cell is surrounded by a perivascular basement membrane and a few spherical mitochondria. Notice the presence of vacuoles.

Fig. 12

Transmission electron micrographs of rats' mammary tumors. DMBA carcinogenesis shows dilated blood vessels with nuclei of its endothelial cells and its perivascular basement membrane showing disorganization. Increased tumor collagen fibers formation. Pyknotic nucleus with chromatin condensation. Note abnormal basal folding is pronounced from the basement membrane. Along the nuclear envelope, nuclei with marginated heterochromatin are visible as a thin, dense line and few spherical mitochondria present. Degenerated cancer cells showing: (1) Cell shrinkage (2) Apoptotic cell with cytoplasmic fragmentation (3) Karyorrhexis nucleus; lysed cytoplasm and dense collagen fibers. PVP-capped AuNRs treated group showing normal endothelial cell lines blood vessel with aggregation of PVP-capped AuNRs within the lumen (arrow). Macrophage cell with an irregularly shaped nucleus and numerous lipid droplets. NIR laser irradiation-treated group showing breast lobule lining with epithelial and myoepithelial cells. Also, observe a few scattered spherical mitochondria in the cytoplasm. two secretory cells with numerous lysosomes. DMBA carcinogenesis treated with PVP-capped AuNRs, and NIR laser irradiation group showing fibroblast cells with extensive cytoplasmic projections and collagen fibers deposition. Myoepithelial cells have lysosomes and lysed mitochondrial cristae, as well as an irregularly shaped nucleus with prominent peripheral chromatin condensation. Normal nucleus with peripheral chromatin condensation, vesicular-swollen mitochondria, and lysosomes.
